# Subchronic Graphene Exposure Reshapes Skin Cell Metabolism

**DOI:** 10.1021/acs.jproteome.2c00064

**Published:** 2022-05-25

**Authors:** Javier Frontiñan-Rubio, Emilio Llanos-González, Viviana Jehová González, Ester Vázquez, Mario Durán-Prado

**Affiliations:** † Faculty of Medicine, Universidad de Castilla-La Mancha, 13071 Ciudad Real, Spain; ‡ Instituto Regional de Investigación Científica Aplicada (IRICA), Universidad de Castilla-La Mancha, 13071 Ciudad Real, Spain; § Faculty of Chemical Science and Technology, Universidad de Castilla-La Mancha, 13071 Ciudad Real, Spain

**Keywords:** graphene, metabolism, skin cells, sublethal, subchronic

## Abstract

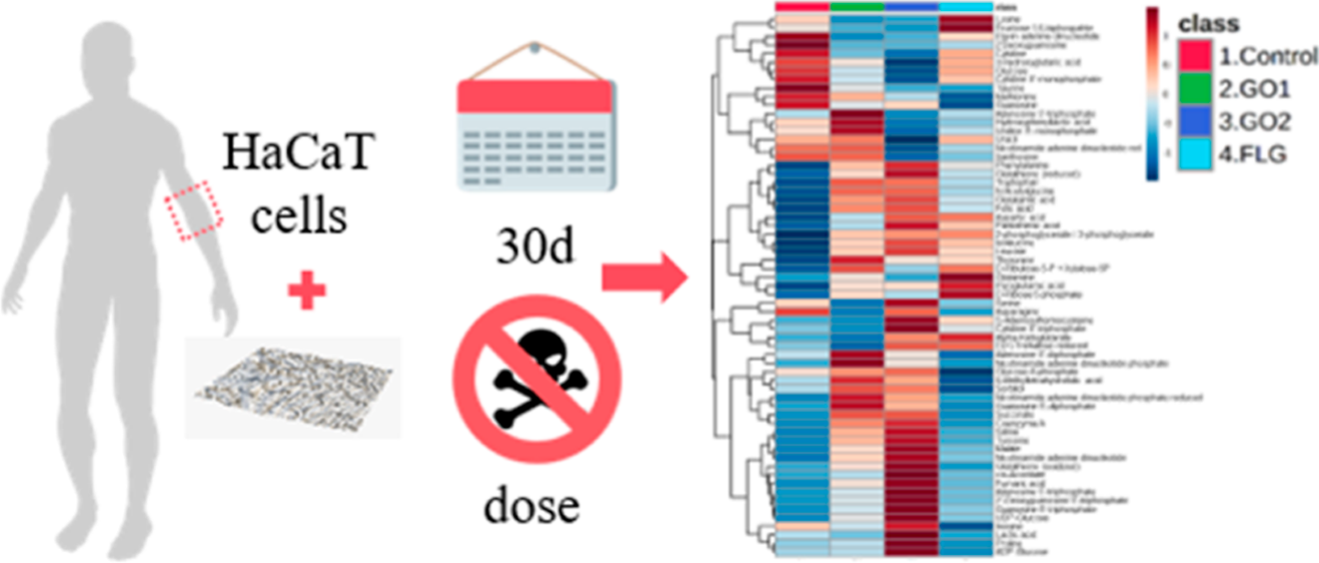

In recent years, the toxicity of graphene-related materials (GRMs) has been evaluated in diverse models to guarantee their safety. In most applications, sublethal doses of GRMs contact human barriers such as skin in a subchronic way. Herein, the subchronic effect (30 day exposure) of three GRMs (GO 1, GO 2, and FLG) with different oxidation degrees and sizes was studied. The effects of these materials on human skin cells, HaCaTs, were assayed through high-throughput metabolic-based readout and other cell-based assays. A differential effect was found between the different GRMs. GO 2 induced a metabolic remodeling in epithelial cells, increasing the level of tricarboxylic acid components, mirrored by increased cell proliferation and changes in cell phenotype. The oxidation degree, size, and method of manufacture of GRMs dictated harmful effects on cell metabolism and behavior generated by nontoxic exposures. Therefore, a “safe by design” procedure is necessary when working with these nanomaterials.

## Introduction

Graphene has emerged as one of the most outstanding nanomaterials due to its extraordinary properties. This two-dimensional material is characterized by a high conductivity, elasticity, and strength, among other attributes, making it a strong candidate for numerous applications.^1−3^ This material is expected to revolutionize different health fields, including drug delivery, medical imaging, and radiotherapy.^1,4−7^ Therefore, it is essential to assess possible adverse health effects, especially in medical applications, smart clothes, or devices that involve contact with the skin and other human tissue barriers or in occupational and environmental domains.^8,9^ It has been highlighted in recent publications that physical (shape, length, and size) and chemical properties are significant factors for graphene-related materials (GRMs).^8,10,11^ Toxicity also depends on the GRM dose, the model used in the assay (e.g., cell line or mice model), and, in particular, on the exposure time used in the experiments.^12−14^ A systematic understanding of GRM-induced alterations is still required, especially at long exposure times, an issue that has hardly been studied to date.

In the last decade, it has been outlined that sublethal doses of GRMs, which induce neither apoptosis nor necrosis, can have a deleterious effect on human cells by impairing cell metabolism and homeostasis.^10,15,16^ Furthermore, alteration of cell metabolism is an essential hallmark of the effect of GRMs on human cells.^17^ However, the question arises about the effect of cell exposure to nontoxic, sublethal doses over prolonged periods. In this regard, metabolomics provides a general overview of the homeostatic state of human cells, the profiling of crucial metabolites as biomarkers, and mechanistic insights into the induced damage.^17−19^ As a whole, cell metabolism and metabolomics identify phenotypic changes that occur in the presence of GRMs and that cannot be perceived with classic cytotoxicity approaches, even more so when the noncytotoxic effect is proven.^20^ As demonstrated in our previous works, NMR-based metabolomics identified relevant metabolic changes in human skin cells exposed to short-term, acute, and nontoxic doses of different GRMs.^15,21^ It is noteworthy that numerous applications of GRMs will involve chronic exposure to low doses,^22−24^ but previous studies have not been carried out to evaluate the effect of such exposure. Therefore, the evaluation of sublethal doses and subchronic exposure is necessary.

The study reported here aims to investigate how subchronic and sublethal exposure of epithelial cells to different well-characterized GRMs affects cell biology. To obtain a complete roadmap of the cell metabolome, we have used ultra-high-performance liquid chromatography–mass spectrometry (UHPLC–MS/MS) to obtain profiles of different metabolites. This approach shows much higher and better sensitivity and resolution than the approaches used in previous related work.^25^ In this regard, HaCaT cells were treated with three different GRMs, namely, a few-layer graphene (FLG) and two commercial graphene oxides (GOs) prepared from different starting materials [carbon nanofibers (GO 1) and graphite (GO 2)]. The materials differed significantly in lateral dimension and size,^10,15^ and a sublethal dose (5 μg/mL) was administered with comparisons made at 7 and 30 days.

The results show a differential effect on cell metabolism after short-term exposure, with the effect being more pronounced for GO treatment than for FLG. In long-term exposures, GO 2, a larger GRM, triggers critical changes in the tricarboxylic acid (TCA) cycle, lately causing a metabolic profile that shares some issues with tumor cells by making cells grow and move more. This effect is differential and depends on the oxidation state and size of the GRM. These findings also highlight the importance of the safety by design approach.

## Experimental Section

### GRM Production and Characterization

GO 1 and GO 2 were obtained from the Antolin group (Burgos, Spain) and Graphenea (San Sebastián, Spain), respectively. GO 1 was produced by the oxidation of carbon fibers (GANF Helical-Ribbon Carbon Nanofibres, GANF) with a KMnO_4_/H_2_SO_4_ mixture and sodium nitrate at 0 °C.^26^ The concomitant carbon debris and other possible acid traces were removed by washing with Milli-Q water, with sequential cycles of redispersion/centrifugation (4000 rpm, 30 min) and discarding the supernatant liquid in each cycle until the pH of the GO 1 aqueous suspension was ∼5. The GO 1 suspension was then freeze-dried.

GO 2 was used as received. FLG was prepared by a ball-milling treatment following the protocol described in the study by González-Domínguez et al.^27^ Briefly, graphite (7.5 mg, purchased from Bay Carbon) and melamine (22.5 mg from Sigma-Aldrich) were ball-milled in a Retsch PM 100 planetary mill at 100 rpm for 30 min. The final powders were dispersed in 20 mL of water and sonicated for 1 min to produce a black suspension. This suspension was dialyzed to remove melamine by dialysis against hot water at 70 °C. Finally, the resulting dispersion was left to settle for 5 days to precipitate. The resulting graphene was extracted from the liquid fraction, freeze-dried, and used as a fine powder.

Typical high-resolution transmission electron microscopy (HRTEM) images of GO 1, GO 2, and FLG are shown in Supporting Information, Figure S1A,B, with graphene flake sizes between 50 nm and 2 μm and their corresponding size distribution with average values of 1.18 μm ± 994 nm, 2.17 μm ± 1.58 μm, and 300 ± 23 nm for GO 1, GO 2, and FLG, respectively. Raman spectroscopy was used to determine the properties of the carbon nanomaterials (Supporting Information, Figure S1C) using their characteristic bands (D, G, and 2D at 1350, 1580, and 2700 cm^–1^, respectively). The G band (around 1580 cm^–1^) is due to the presence of sp^2^ carbon atoms in the hexagonal structure. The 2D band (around 2700 cm^–1^) represents the quality of carbon rings in the graphene layers,^28^ and it was also used to determine the number of layers (*N*_G_) in FLG using the equation reported in the study by Coleman et al.^26^ An average of three layers was calculated for FLG. GO 1 and GO 2 showed a 2D band of low intensity due to the high structural defects in the carbon rings.^29^ The intensity ratio between the D (around 1350 cm^–1^) and G bands (*I*_D_/*I*_G_) is used to quantify the density of defects,^30^ with values of 0.94, 0.75, and 0.42 obtained for GO 1, GO 2, and FLG, respectively. Thermogravimetric analysis (TGA; Supporting Information, Figure S1D) of GO 1, GO 2, and FLG showed weight losses of 37.86, 43.66, and 4.94% at 500 °C, respectively. GO 2 showed the highest mass loss in the range of 100–300 °C, and this is attributed to the decomposition of functional groups (−OH, −COOH, and −C–O–C)^31,32^ and the remaining stable oxygenic functional groups (e.g., esters).^31,32^ Finally, elemental analysis of the materials (Supporting Information, Figure S1E) showed a similar percentage of oxygen (48–49 wt %) for GO 1 and GO 2 but only 6.53% for the FLG sample. These values are consistent with the results of the TGA.

### Cell Culture

HaCaT cells from a spontaneously immortalized human keratinocyte line were maintained in Dulbecco’s modified Eagle’s medium (DMEM) (Sigma-Aldrich) supplemented with 10% fetal bovine serum (FBS) (Sigma-Aldrich) and 1% antibiotic/antimycotic (Sigma-Aldrich) at 37 °C in a 5% CO_2_ atmosphere. Cells were used up to the 15th passage.

### Exposure of Cells to GRMs

Cells were exposed to different GRMs for 7 days (Supporting Information, Figure S2A) or 30 days (Supporting Information, Figure S2B). Cell cultures were maintained according to standard procedures. Cells received a fresh medium every 3–4 days and were subcultured and treated with GRMs (5 μg/mL) every 7 days (Supporting Information, Figure S2).

### Necrosis and Apoptosis Assays

Necrosis and apoptosis assays were performed following the protocol reported previously by our research team.^15^ Briefly, after different treatments, HaCaT cells were seeded in 96-well plates, and after 24 h, cells were treated with 10 μg/mL EtBr (Sigma-Aldrich) and 1 μM Calcein-AM (Thermo Fisher) for 30 min. Viable (green) and necrotic (red) cells were determined by fluorescence microscopy using a Cytation 5 system (BioTek). Image analysis was also conducted using ImageJ software (ImageJ). Immediately after image acquisition, cells were fixed and permeabilized for 2 min in ice-cold methanol and stained with 1 μg/mL Hoechst (Sigma-Aldrich). Apoptotic nuclei were determined according to morphological criteria.^15^ Data are presented as the percentage of necrotic or apoptotic cells versus the total (*n* = 3).

### Sample Preparation and Measurements for Metabolomics

HaCaT cells were incubated with GO 1, GO 2, or FLG for 7 or 30 days (five samples per treatment and time). Cells were harvested in a tube using a cell scraper, centrifuged (4 min at 1000 rpm), and resuspended and washed in 1 mL of phosphate-buffered saline (PBS). The cell mixture was transferred into Eppendorf tubes and centrifuged again (4 min at 1000 rpm); the supernatant was removed, and the cell pellet was frozen at −80 °C. The same procedure was performed with the same supplemented media without cells as blank samples in parallel. Metabolite extraction was accomplished by fractionating keratinocytes into pools of species with similar physicochemical properties using appropriate combinations of organic solvents. As described by Barr et al.,^33,34^ the following method was used according to the chemical class of the target analytes. HaCaT cells were defrosted on ice, and proteins were precipitated from the lysed cell samples by adding the extraction solvent spiked with metabolites not detected in unspiked cell extracts (internal standards). Cell extracts were then incubated at −20 °C for 1 h, and samples were vortexed and centrifuged at 18,000*g* for 10 min at 4 °C. Supernatants were collected and kept on ice. A second extraction was performed on the remaining pellets following the steps described above. Supernatants obtained from the second extraction were collected and combined with the supernatants of the first extraction. Finally, these supernatants were dried under vacuum, reconstituted in water, resuspended with agitation for 10 min, centrifuged at 18,000*g* for 5 min at 4 °C, and transferred to vials for UHPLC–MS analysis. Randomized sample injections were performed, with each quality control (QC) calibration and validation extract uniformly interspersed throughout the entire batch run. Specific metabolite extraction procedures, chromatographic separation conditions, and mass spectrometric detection conditions are also detailed in ref (34). Metabolomic analyses were performed by OWL Metabolomics (Bizkaia, Spain).^34^ Briefly, chromatography was performed on an Acquity HSS T3 1.7 μm column (Waters Corp., Milford, MA) using an ACQUITY UPLC system (Waters Corp.). The eluent was introduced into the mass spectrometer LCT Premier (Waters Corp.) by electrospray ionization, with capillary and cone voltages set in the negative ion mode.

### Metabolomics Data Processing

Data preprocessing generated a list of chromatographic peak areas for the metabolites detected in each sample injection. Data normalization was performed following the procedure described in the study by Martínez-Arranz et al.^35^ First of all, the different metabolites were identified, and the determination was carried out using Waters QTOF Premier and Xevo G2 (Waters Corp., Milford, MA) instruments. LC–MS features (as defined by the retention time, mass-to-charge ratio pairs, and *R*_t_-*m*/*z* were associated with identified metabolites by comparison of their accurate mass spectra and chromatographic retention times in the extracts with those obtained using available reference standards (three different mixtures of standards were used) (Supporting Information, Table S1). A metabolic feature with a *m*/*z* value between 400 and 1000 Da was considered unambiguously identified when *R*_t_ difference with respect to the standard was smaller than 2 s and the deviation from its *m*/*z* value (δ_m/z_) was smaller than 3 ppm. For metabolites with *m*/*z* values smaller than 400 Da, the criterion followed with respect to *R*_t_ was the same, but the δ_m/z_ limit was set to 1.2 mDa.

Once normalized, the dimensionality of the complex data set was reduced to enable easy visualization of any metabolic clustering of the different groups of samples. This was achieved by multivariate data analysis, including nonsupervised principal component analysis (PCA) (Supporting Information, Figure S3). Univariate statistical analyses were also performed by calculating group percentage changes and the unpaired Student’s *t*-test *p*-value (or Welch’s *t*-test where unequal variances were found). Other metabolic analyses, including metabolic visualization (i.e., heat map) and enrichment analysis, were performed using MetaboAnalyst 4.0.^36^

The dataset and raw files are available at the NIH Common Fund’s National Metabolomics Data Repository website, the Metabolomics Workbench, www.metabolomicsworkbench.org (ID ST002157).

### Cellular Energetics

The Seahorse XFp analyzer (Seahorse Biosciences, North Billerica, MA) was used to measure the oxygen consumption rate (OCR) and extracellular acidification rate (ECAR) following the protocol set up previously by Divakaruni et al.^37^ Briefly, after long-term treatment, HaCaT cells were incubated in the XFp base medium (Seahorse Biosciences, North Billerica, MA) [with 1 mM pyruvate, 2 mM glutamine, and 10 mM glucose (Sigma-Aldrich)] using a density of 3 × 10^5^ cells per well in Seahorse XFp miniplates. Plates were incubated for 60 min at 37 °C without CO_2_ and loaded into the Seahorse analyzer. For cell energy phenotype determination, three baseline OCR and ECAR measurements were taken for each well within the first 20 min, and then, a mixture with oligomycin (1 μM) and Carbonyl cyanide 4-(trifluoromethoxy)phenylhydrazone (FCCP) (0.3 μM) was injected. Furthermore, three OCR and ECAR values were automatically calculated. Data are presented as mean ± standard error of the mean (SEM) for each time point in pmol/min normalized according to the number of cells per well. For normalization, cells were fixed and permeabilized for 2 min in ice-cold methanol and then stained with 1 μg/mL Hoechst. The number of cells per well was obtained using a Nikon TiU epifluorescence microscope with a 2× objective and counted with ImageJ. Data are presented as mean ± SEM for each time point in pmol/min normalized to the number of cells per well (*N* = 3).

### Ki-67 Immunolabeling

Ki-67 positive cells were assayed by immunocytochemistry using a specific monoclonal antibody (Santa Cruz BT). Briefly, cells treated for 30 days were seeded in 96-well plates. The medium was removed, and cells were fixed for 2 min in cold methanol, blocked, and incubated for 60 min with an anti-Ki-67 antibody (1:500). The cells were then stained with an AlexaFluor-594 anti-mouse dye (Invitrogen) for 60 min. Images were acquired on a Cytation 5 Reader (BioTek) using the 20× objective and analyzed with ImageJ.

### Colony Formation Assay

Cells were incubated with GO 1, GO 2, or FLG for 30 days and were then seeded at 200 cells/well in 24-well plates. After 14 days, cells were fixed for 2 min in cold methanol and stained with 0.01% (w/v) crystal violet for 30 min. Plates were dried, and colonies containing more than 50 individual cells were determined using bright-field microscopy (2×) on a Cytation 5 Reader (BioTek).

### Wound Healing Assay

The wound healing assay was carried out by the protocol set up previously.^15^ HaCaT cells were incubated with GO 1, GO 2, or FLG for 30 days. After each treatment, cells were plated in 24-well plates, cultured to confluence, and then serum-starved for 12 h. A cross-scratch was done in the monolayer with a 200 μL pipette tip, and a fresh medium replaced the medium. Images were acquired on a Cytation 5 Reader (BioTek) using a 4× objective. The percentage of wound closure was calculated by measuring the open area free of cells for each image, using ImageJ, immediately after making the scratch and 48 h after treatment. The results shown are an average of *n* = 3.

### Nuclear and Cell Size Study

HaCaT cells were incubated with GO 1, GO 2, or FLG for 30 days. After treatment, cells were plated in 96-well plates and stained with Hoechst 33342 solution (Thermo Fisher). Bright-field and Hoechst images were acquired on a Cytation 5 Reader (BioTek) using a 20× objective and analyzed with ImageJ (>50 cells/treatment).

### Statistical Analysis

Data are expressed as mean ± SEM for three independent experiments (*n* = 3). Statistical analysis was carried out using GraphPad Prism 8 (San Diego, CA, USA) using Student *t*-test or one-way analysis of variance (ANOVA) followed by Bonferroni’s post-test. Significance levels were considered at *p* < 0.05.

## Results

### Characterization of GRMs

Supporting Information, Figure S1A, shows the typical HRTEM images of GO 1, GO 2, and FLG with graphene flakes in the size range between 50 nm and 2 μm, and their corresponding distribution sizes are given in the Supporting Information, Figure S1B, with an average size of 1.18 μm ± 994 nm, 1.45 μm ± 637 nm, and 300 ± 23 nm for GO 1, GO 2, and FLG, respectively. Raman spectroscopy was used to determine the properties of these carbon nanomaterials (Supporting Information, Figure S1C) with their characteristic bands (D, G, and 2D at 1350, 1580, and 2700 cm^–1^, respectively). The D band is related to some amorphous phases in the carbon rings of graphene structures. The G band is due to sp^2^ carbon bonds in the hexagonal structure. The 2D band represents the quality of carbon rings in the graphene layers.^28^ The 2D band was also used to determine the number of layers (*N*_G_) in FLG using the equation described by Coleman et al.^26^ We calculate an average of three layers with a full width at half-maximum (FWHM) of 65.63 cm^–1^ for FLG. Meanwhile, GO 1 and GO 2 show a low intensity in the 2D band due to the high structural defectiveness of carbon rings in the graphene layers of the nanomaterials.^29^ The intensity ratio between D and G bands (*I*_D_/*I*_G_) quantifies the density of defects in graphene,^30^ giving values of 0.94, 0.75, and 0.42 for GO 1, GO 2, and FLG, respectively. TGA (Supporting Information, Figure S1D) of GO 1, GO 2, and FLG was performed under a nitrogen atmosphere showing a weight loss due to the residual oxygen-containing groups at the edges of the graphene sheets of 57.30, 55.05, and 4.81% at 600 °C, respectively. GO 1 and GO 2 show the major mass loss in the range of 100–300 °C, attributed to the decomposition of functional groups (−OH, −COOH, and −C–O–C)^31,32^ and the remaining stable oxygenic functional groups (e.g., esters).^31,32^ Finally, elemental analysis of GO 1, GO 2, and FLG (Supporting Information, Figure S1E) shows a 48–49 wt % of oxygen groups in the samples GO 1 and GO 2 and only 6.53% in the sample of FLG. These values are in concordance with the values observed in TGA.

### Toxicity of Long-Term GRM Exposure

Several previous studies have concerned about the cytotoxicity and other cellular alterations induced by different GRMs in many human cell lines.^8,15,38−40^ Nevertheless, there is a lack of information about the detrimental effects of chronic and subchronic GRM exposure. Therefore, after characterization of the three GRMs (Supporting Information, Figure S1), we explored the possible cytotoxic effects of the compounds at low doses.

**Figure 1:**
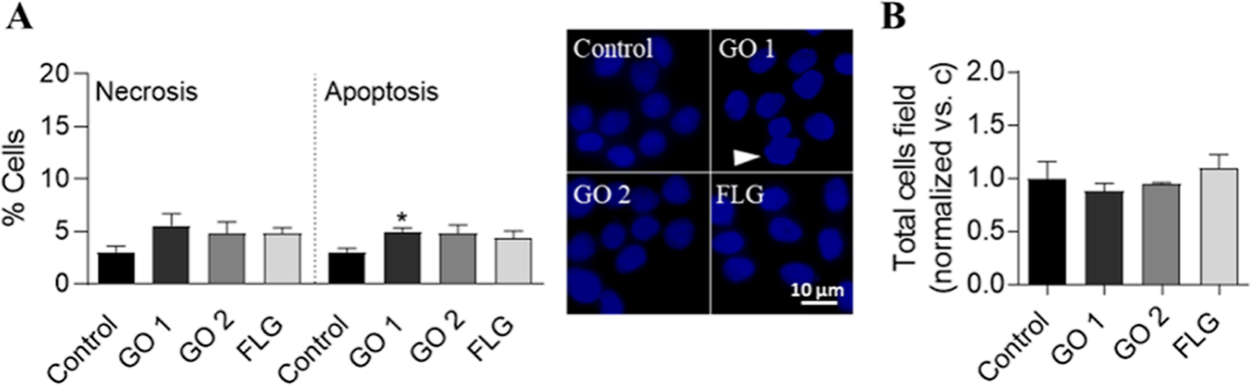
Effect of GRMs on HaCaT cell necrosis, apoptosis, and viability. (a) Percentage of necrotic (left graph) and apoptotic (right graph and pictures) cells and (b) normalized number of HaCaT cells per field, treated with a 5 μg/mL dose of GO 1, GO 2, and FLG for 30 days. Graphs represent mean ± SEM. *p < 0.05 (*N* = 3).

The effect of a sublethal dose of 5 μg/mL GO 1, GO 2, and FLG on cell viability was assessed along with necrosis and apoptosis upon exposure for 30 days. The different GRMs induced a nonsignificant increase in necrosis and a slight rise in apoptosis (Figure 1A, white arrow) induced by GO 1 compared to control cells (*p* < 0.05). Alterations were observed in terms of viability (Figure 1B), and therefore, the different GRMs hardly generate cytotoxicity at a dose of 5 μg/mL.

Acute 1 week treatments with nontoxic doses of GRMs altered the metabolite profiles of epithelial cells.

**Figure 2:**
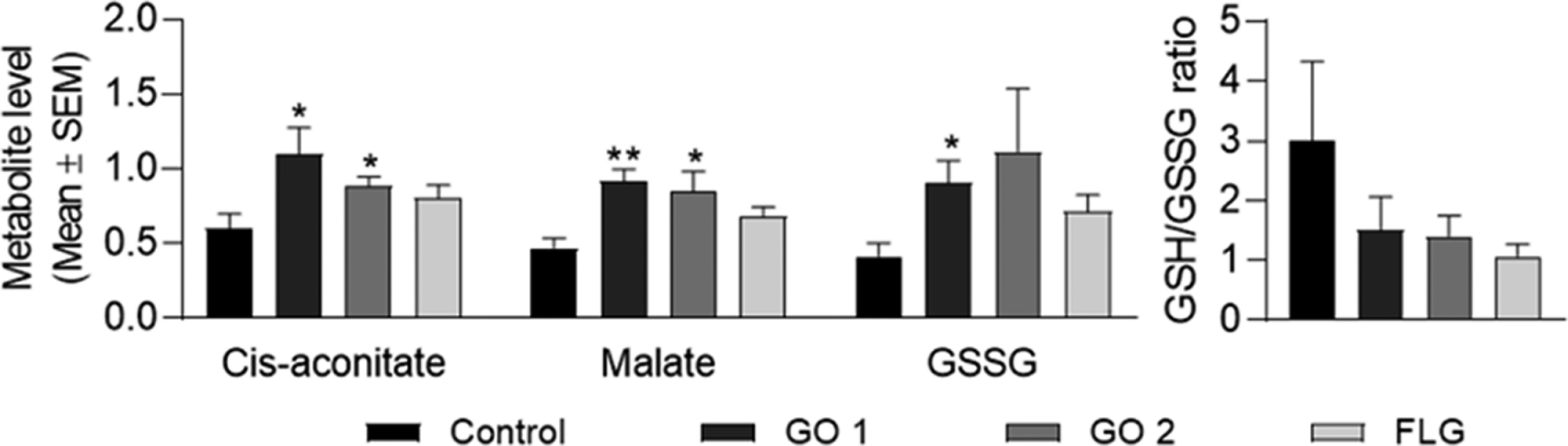
Effect of acute treatments with nontoxic doses of GRMs on HaCaT cell metabolites. Normalized levels of different relevant metabolites in cells treated for 1 week with 5 μg/mL GO 1, GO 2, or FLG. Graphs represent mean ± SEM. *p < 0.05; **p < 0.01; *N* = 5. Subchronic treatments with nontoxic doses of GRMs altered the metabolome of epithelial cells.

Metabolomics is an emerging tool that enables the detailed characterization of metabolic phenotypes and remodeling of pathways.^17,20,41−44^ The impact of GRMs on the HaCaT’s metabolome was evaluated by MS coupled with UHPLC. A differential effect between GOs and FLG was observed in cells treated for 7 days. GO 1 and GO 2 led to an increase in six amino acids, two carboxylic acids, and one nucleotide and a decrease in cytidine levels (Supporting Information, Table S2 and Figure S4). GO 1 boosted critical metabolites such as oxidized glutathione (GSSG), NADP, NADPH, or fumaric acid, while GO 2 diminished flavin adenine dinucleotide (FAD) levels (Supporting Information, Table S2 and Figure S4). FLG led to a slight increase in malate, succinate, and GSSG (Supporting Information, Table S2 and Figure S4). Despite having different lateral sizes, the two GOs evaluated had a similar impact on metabolism and differed from that of FLG. It is essential to highlight the increase in the levels of *cis*-aconitate and malate when compared to control cells (Figure 2). These two metabolites, that is, *cis*-aconitate and malate, are essential components of the TCA cycle, one of the main pathways for normal (aerobic) energy metabolism.^45^ Therefore, even without evidence of cytotoxicity in acute treatment, the more oxidized graphene had a greater impact on metabolism. The results also show an increase in the level of GSSG in cells treated with GO 1 (Figure 2, p < 0.05) that did not reach significance with GO 2 or FLG (Figure 2).

**Figure 3:**
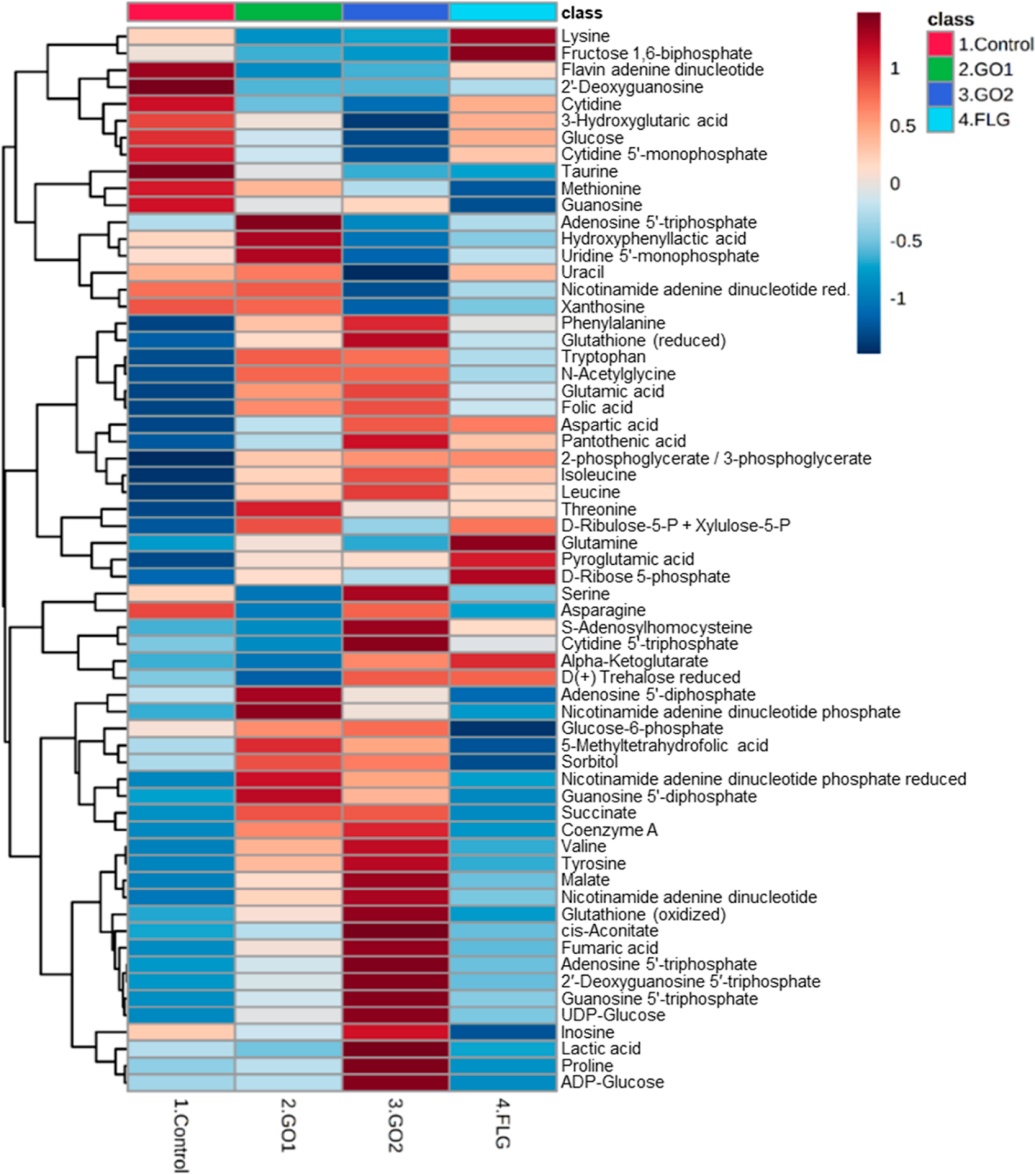
Metabolites altered in HaCaT cells treated for 30 days with GRMs. Heatmap and clustering of metabolites corresponding to HaCaTs treated with 5 μg/mL GO 1, GO 2, or FLG for 30 days. Each class is shown as an average of *n* = 5.

The main goal of the work described here was to analyze the effect of long-term subchronic exposure on epithelial cells of GRMs and ascertain how metabolism was modified. GO 1 affected the levels of 3 metabolites, whereas GO 2 altered 17 and FLG only 2 (Figure 3 and Supporting Information, Table S3 and Figure S5). Enrichment analysis revealed a significant impact of GO 2-treated cells (Supporting Information, Figure S6) on key metabolic pathways such as cellular bioenergetics and amino acid metabolism. As shown by enrichment analysis, GO 1 and FLG had a lower impact on the different metabolic pathways (Supporting Information, Figures S7 and S8).

**Figure 4:**
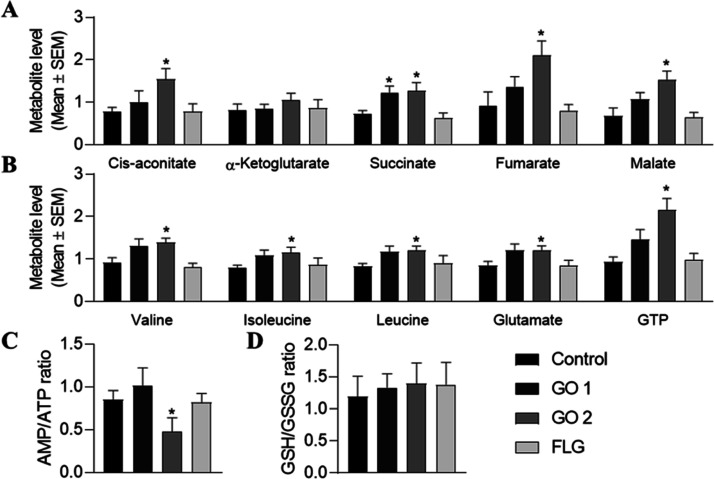
Effect of subchronic treatments with nontoxic doses of GRMs on HaCaT cell metabolites. Normalized levels of different relevant metabolites in cells treated for 30 days with 5 μg/mL GO 1, GO 2, or FLG. Normalized levels of (a) TCA cycle components and (b) BCAAs and GTP, (c) AMP/ATP ratio, and (d) GSH/GSSG ratio. Graphs represent mean ± SEM. *p < 0.05, *N* = 5. Subchronic treatments with nontoxic doses of GRMs altered the bioenergetics of epithelial cells.

The Krebs or TCA cycle is the primary source of cellular energy and provides precursors for different biosynthetic pathways.^45^ The cycle produces intermediates for use as building blocks in the synthesis of macromolecules and energy and electron acceptors.^46^ GO 1 elevated the succinate level, indicating alterations in the TCA cycle and cellular bioenergetics (Figures 3 and 4A). Enrichment analysis showed an impact on this pathway and other changes related to energetics (Supporting Information, Figure S6). GO 2 increased five of the TCA cycle main components: *cis*-aconitate, succinate, fumarate, malate (Figures 3 and 4A), and α-ketoglutarate, with the latter not reaching significance, which overall translates to a decrease in the AMP/ATP ratio (Figure 4C). FLG did not alter any TCA cycle component (Figures 3 and 4A). Treatment with GOs enhanced the level of branched-chain amino acids (BCAAs), leucine, isoleucine, and valine, with this effect being more pronounced with GO 2 (Figure 4B). FLG did not affect the BCAA levels (Figure 4B). GOs, mainly GO 2, altered the levels of other intermediate metabolites in the TCA cycle, such as glutamate and GTP (Figure 4B).

Conversely, FLG downregulated two metabolites, that is, 5-methyltetrahydrofolate (5-MTHF) and FAD, with the latter also being diminished by GO 1 and GO 2 (Figure 3 and Supporting Information, Table S3 and Figure S5).

**Figure 5:**
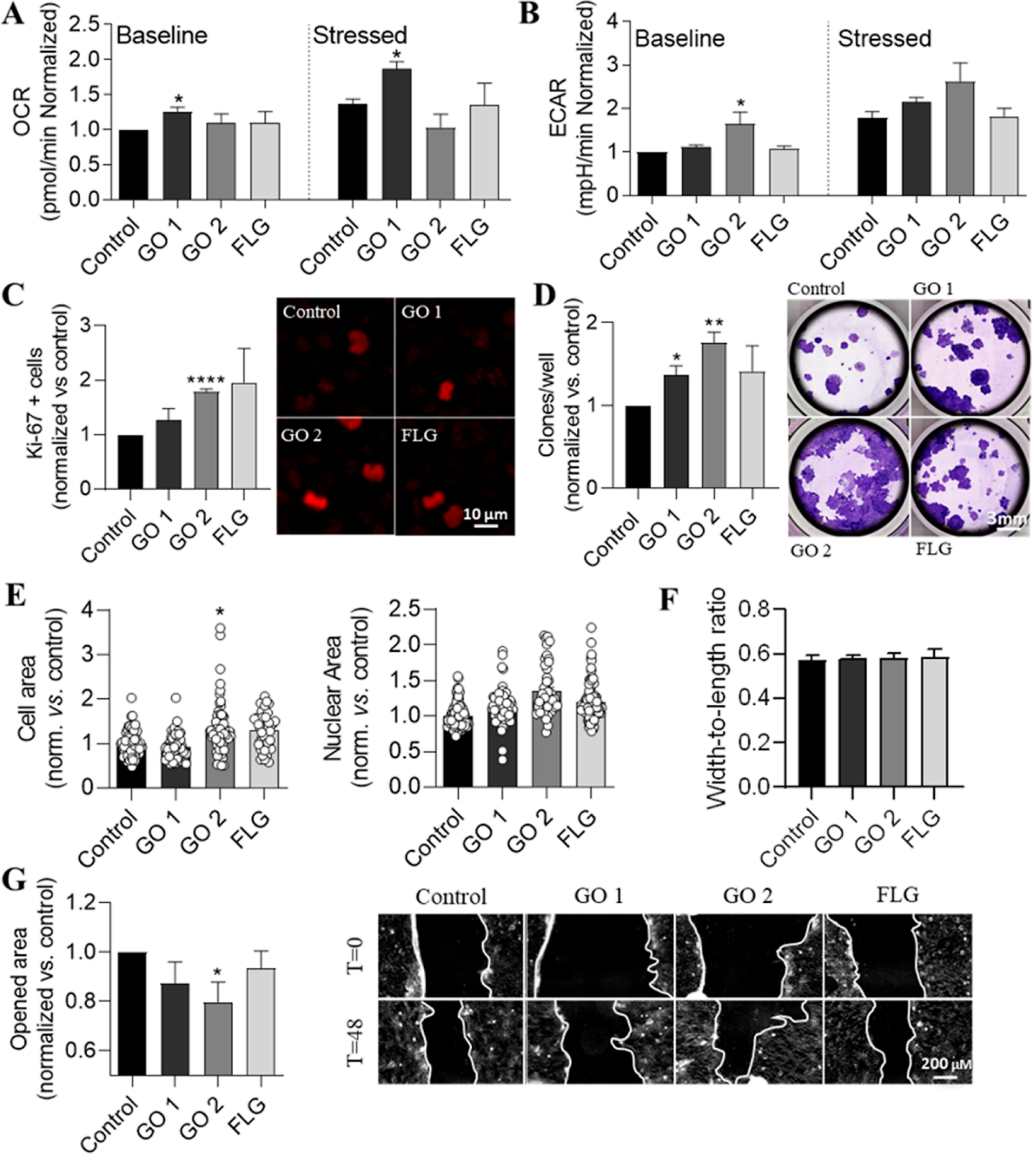
Effect of subchronic treatments with nontoxic doses of GRMs on HaCaT cell bioenergetics, proliferation, phenotype, and motility. Levels of (a) OCR cell respiration and (b) ECAR glycolysis under basal and stressed conditions. (c) Proliferation was measured as the number of Ki-67+ cells and (d) colony formation assay by limiting dilution. (e,f) Distribution of cell phenotype based on the nuclear and cell areas and cell shape, measured as cell width-to-length ratio. (g) Wound healing assay (>50 cells) at 48 h. Cells were treated for 30 days with 5 μg/mL GO 1, GO 2, or FLG. Graphs represent mean ± SEM, *n* = 3; normalized vs control, *p < 0.05; ****p < 0.0001.

To gain a greater insight into how cellular bioenergetics are affected by nontoxic doses of GRMs, HaCaT cells exposed to 5 μg/mL GO 1, GO 2, and FLG for 30 days were analyzed using Seahorse XFp Extracellular Flux.^37^ This tool allowed the OCR to be quantified as a measure of mitochondrial respiration and the ECAR as a measure of glycolysis in living cells.^37^ The adenosine triphosphate (ATP) synthase oxygen consumption mainly measures mitochondrial respiration. Glycolysis is quantified by measuring the ECAR of the surrounding medium, which arises from the excretion of lactic acid per unit time after its conversion from pyruvate (Supporting Information, Figure S9).^47,48^ Energy phenotype tests based on the OCR and ECAR of the cells were performed to determine the energetic phenotype under baseline and stressed (energy demand) conditions. GO 1 increased the OCR, which was maintained in an energy demand situation in the presence of the stressor compounds oligomycin and FCCP, whereas GO 2 and FLG did not affect the OCR (Figure 5A). GO 2 increased lactate levels (Supporting Information, Figures S9 and S10), and this was mirrored by a significant increase in glycolytic function, which was measured as the ECAR (Figure 5B).

Subchronic treatments with nontoxic doses of GRMs increased proliferation, induced phenotypic changes, and enhanced motility of epithelial cells.

Uncontrolled proliferation is one of the major hallmarks of cancer.^49,50^ Ki-67 is a widely used and approved cellular marker for proliferation,^51^ and this marker was therefore evaluated in HaCaT cells treated with GRMs. The results show an increase in the overall nuclear Ki-67 intensity in cells exposed to the different GRMs (Supporting Information, Figure S11A), and this is more evident upon considering the number of Ki-67 positive cells (signal above a threshold). Among the GRMs, GO 2 was more potent than GO 1 or FLG (Figure 5C).

To gain further insights into proliferation, limiting dilution-based clonogenic assays were performed to assess the ability of a single cell to grow and form a colony. GRMs—mainly GO 2—increased the number of colonies (Figure 5D).

The tumor transformation process involves some cell phenotypic changes.^50^ Larger nuclear size “atypia” is a hallmark of cancer cells, and it is related to metastasis, migration, and proliferation, among other changes.^52,53^ The whole cell and nuclear sizes of HaCaT incubated for 30 days with different GRMs were evaluated. An increase in both the nuclear and cell sizes was found, and this effect was more marked for GO 2 (Figure 5E,F and Supporting Information, Figure S11B). A classical wound healing assay assessed the ability of cells exposed to GRMs to move in two-dimensional surfaces. This strategy showed a reduction in the open area in cells treated with GO 2, which indicates enhanced cell motility (Figure 5G). GO 1 and FLG did not affect motility (Figure 5G). This finding correlates with the accumulation of oncometabolites, another hallmark of cancer.^54^

## Discussion

HaCaT cells have become the prototype model for skin toxicity due to their resemblance to epidermal keratinocytes.^55,56^ Moreover, these epithelial cells have been extensively used in GRM-induced toxicity studies.^10,15,38,57,58^ Our previous work shows that exposure of HaCaT cells to GRMs induced a dose-dependent decrease in viability at short incubation times (24 h, 72 h, or 1 week), being the threshold at 5 μg/mL.^10,15^ Thus, it is essential to obtain more information about the effect of GRMs on the biology of epithelial cells. In this regard, metabolomics offers a complete scenario of how cells are affected, and this can be complemented by other approaches.^17,20^ The effect of sublethal doses in contact with cells for long periods should be the primary concern before GRMs are used for any commercial application as these compounds can be quickly taken up by epithelial cells, and they can reach mitochondria and nuclei in a few hours.^59^ Despite this, there are hardly any publications on the subject.

Three GRMs that differ in the oxidation state and lateral size were characterized, namely, GO 1, GO 2, and FLG. GO 1 and FLG have similar sizes but differ in the oxidation state. GO 2 is oxidized to a similar extent to GO 1, but it is a larger GRM (Supporting Information, Figure S1). The results presented herein indicate that a 7 day exposure damaged energy metabolism and increased cellular oxidative stress (OE), leading to cell death upon longer exposures. It has previously been reported that graphene induction of cell death is mediated by increased OE.^15,60,61^ This behavior seems to be related to an alteration of key metabolites such as reduced glutathione (GSH), one of the primary cellular antioxidants.^62^ The ratio of GSH to GSSG indicates cellular wellness.^62^ Besides, our results provide evidence that GRMs decreased the GSH:GSSG ratio (Figure 2), suggesting that even though cells are not dead, they may be compromised.

The possibility outlined above is reinforced by the results obtained by metabolomics at longer subchronic incubation times. GRMs, mainly GO 2, affect the TCA cycle, and this is an attempt by the cell to improve its energy capacity.^45^ The increases in the levels of succinate and fumarate triggered by GO are worth highlighting because they are known oncometabolites^54^ and can alter the epigenome and lead to tumorigenesis.^63^ The accumulation of fumarate and succinate stabilizes HIF1α, a key component of the hypoxic tumor response, induces DNA damage, enhances glycolysis, and increases cell proliferation.^54,64−66^ Therefore, there is correlation between the changes observed in GO 2-treated cells (increased oncometabolites, cell proliferation, and glycolysis). On the other hand, the increased amino acid levels could be related to higher cell proliferation in both normal and tumor cells.^67^ GO 2 significantly increased the levels of different amino acids and in turn cell proliferation and clonogenic growth. In particular, the increase in BCAAs (leucine, isoleucine, and valine) is striking in the present study. According to previous publications, the reprogramming of BCAAs is common in different types of tumors, increasing cell growth directly (as protein bricks) and indirectly through the activation of regulators such as mTOR.^67−70^ Moreover, under proliferating conditions, BCAA degradation can be suppressed, leading to an accumulation.^71^

Regarding energetic metabolism, we report an increase in the OCR triggered by GO 1, which does not indicate mitochondrial damage but represents an overactivation that could compromise cellular homeostasis if maintained for long periods. GO 2 reduced the AMP/ATP ratio and increase ECAR levels; these results, together with those mentioned above, led us to hypothesize a general metabolic alteration of cells exposed to this GRM, which would lead to a scenario resembling tumor metabolism. Besides, enhanced glycolysis and ATP levels without affectation of the OCR resemble the Warburg effect, a metabolic alteration observed in tumor cells that increases ATP production by glycolysis and increases the fermentation of glucose to lactate in normoxic conditions.^72,73^ This process is also induced by an accumulation of succinate.^74,75^

Outstandingly, most of these alterations were observed to a lesser extent in GO 1-treated cells and were barely observed in FLG-treated cells. In this sense, FLG just induces a significant decrease in 5-MTHF, which could be associated with alterations in DNA methylation, leading to cell reprogramming.^76^ However, there is no correlation with other results from functional experiments. Therefore, the size and especially the oxidation degree are crucial for the long-term deleterious effect of low doses of graphene on skin cells.

It has been reported in some recent publications that short-term exposure of cells to different GRMs induces DNA damage, cell cycle arrest, proliferation, and other tumor-like phenotypes.^77−79^ Therefore, if these alterations are maintained under a chronic exposure to nontoxic, low concentrations of GRMs, the endpoint could be cell transformation and tumorigenesis. Studies of the subchronic exposure of lung cells to different carbon nanotubes showed potential carcinogenicity.^80^

It is critical to determine whether GRMs can generate a similar effect and how the oxidation degree and lateral size affect this behavior. Proliferative cells require energy and the biosynthesis of nucleotides, proteins, and lipids.^81^ Metabolomics indicated that subchronic exposure of skin cells to GO 2 increased ATP, GTP, dGTP, and different amino acids such as BCAAs (Supporting Information, Table S3), which are essential nutrients that act as a source of energy for tumors.^82^ This situation supports the increase in cell proliferation induced by GO 2.

In this work, we studied the metabolism of human skin cells exposed to sublethal doses of GRMs in acute (7 days) and subchronic (30 days) ways using UHPLC–MS-based metabolomics. GOs and FLG, which have different oxidation states and lateral sizes, induced a differential effect on cellular metabolism—behavior that has already been observed in previous works. However, dramatic metabolic remodeling was observed after a 30 day exposure to GRMs, mainly GO 2. GO 2 is characterized by the largest lateral dimension and an average size due to flakes larger than 2 μm, which are hardly present in the other two materials studied.^10^ These findings indicate that the physicochemical properties of GRMs, especially the oxidation state and size, influence their effect on skin cells. One of the materials studied, GO 2, could be especially harmful as cells treated subchronically with this compound could behave as tumor-prone cells, as indicated by the metabolite profile, metabolic behavior, and their increased growth rate and ability to move.

It is necessary to investigate the cellular mechanisms triggered by GRMs in greater detail, but it is also mandatory to accurately check the tumorigenic potential of these compounds, an aspect that has not been correctly evaluated to date.

## Supporting Information Available

List of reference standards; most relevant metabolites (7 d); most relevant metabolites (30 d); characterization of GO 1, GO 2, and FLG; GRM exposure approach; score scatter plot of the PCA model of HaCaT cells; metabolites altered in HaCaT treated for 7 d with GRMs; metabolites altered in HaCaT treated for 30 d with GRMs; enrichment analysis GO 2 versus control cells (30 d); enrichment analysis GO 1 versus control cells (30 d); enrichment analysis FLG versus control cells (30 d); general metabolism scheme and Seahorse XFp source measurements; effect of GRMs on cell metabolism; and the effect of GRMs on cell proliferation and phenotype (PDF)
