# Supplementary material for: Subchronic Graphene Exposure Reshapes Skin Cell Metabolism
Source: J Proteome Res. 2022 May 25;21(7):1675–85. doi: 10.1021/acs.jproteome.2c00064 (PMC9251767; doi:10.1021/acs.jproteome.2c00064)
Supplement: Supplementary file 1 — pr2c00064_si_001.pdf [file pr2c00064_si_001.pdf]

Subchronic Graphene Exposure Reshapes Skin Cell Metabolism

Javier Frontiñan-Rubio [1], Emilio Llanos-González [1], Viviana Jehová González [2,3], Ester Vázquez [2,3] * and Mario Durán-Prado [1] *.

[1] Universidad de Castilla-La Mancha, Faculty of Medicine, 13071, Ciudad Real, Spain; [2] Universidad de Castilla-La Mancha. Instituto Regional de Investigación Científica Aplicada (IRICA), 13071, Ciudad Real, Spain; [3] Universidad de Castilla-La Mancha Faculty of Chemical Science and Technology, 13071, Ciudad Real, Spain

* Ester Vázquez (ester.vazquez@uclm.es) and Mario Durán-Prado (mario.duran@uclm.es)

S-2,3. Supplementary Table 1. List of reference standards.

S-4,5. Supplementary Table 2. Most relevant metabolites (7d).

S-6,7. Supplementary Table 3. Most relevant metabolites (30d).

S-8. Supplementary Figure 1. Characterization of GO 1, GO 2, and FLG.

S-9. Supplementary Figure 2. GRMs exposure approach.

S-10. Supplementary Figure 3. Score scatter plot of the PCA model of HaCaT cells.

S-11. Supplementary Figure 4. Metabolites altered in HaCaT treated for 7 d with GRMs.

S-12. Supplementary Figure 5. Metabolites altered in HaCaT treated for 30 d with GRMs.

S-13. Supplementary Figure 6. Enrichment Analysis GO2 vs. control cells 30d.

S-14. Supplementary Figure 7. Enrichment Analysis GO1 vs. control cells 30d.

S-15. Supplementary Figure 8. Enrichment Analysis FLG vs. control cells 30d.

S-16. Supplementary Figure 9. General metabolism scheme and Seahorse XFp source measurements.

S-17. Supplementary Figure 10. The effect of GRMs on cell metabolism.

S-18. Supplementary Figure 11. The effect of GRMs on cell proliferation and phenotype.

Reference Standards	Common Name	Formula	m/z	Adduct
Mix 1	Fructose-1,6-bis-P	C6H14O12P2	338,9882	[M-H]-
Mix 1	2,3-bisphosphoglycerate	C3H8O10P2	264,9514	[M-H]-
Mix 1	2-phosphoglycerate / 3-phosphoglycerate	C3H7O7P	184,9851	[M-H]-
Mix 1	Adenosine 5´-diphosphate	C10H15N5O10P2	426,0216	[M-H]-
Mix 1	Adenosine 5´-triphosphate	C10H16N5O13P3	505,9879	[M-H]-
Mix 1	Adenosine 5´-monophosphate	C10H14N5O7P	346,0553	[M-H]-
Mix 1	Glucose	C6H12O6	179,0556	[M-H]-
Mix 1	Glyceraldehyde-3-phosphate	C3H7O6P	168,9902	[M-H]-
Mix 1	D-Fructose-6-phosphate	C6H13O9P	259,0218	[M-H]-
Mix 1	Glucose-6-phosphate	C6H13O9P	259,0218	[M-H]-
Mix 1	Xylulose-5P	C5H11O8P	229,0113	[M-H]-
Mix 1	D-Ribose 5-phosphate	C5H11O8P	229,0113	[M-H]-
Mix 1	Nicotinamide adenine dinucleotide phosphate	C21H28N7O17P3	742,0676	[M-H]-
Mix 1	Nicotinamide adenine dinucleotide phosphate reduced	C21H29N7O17P3	744,0837	[M-H]-
Mix 1	D-Ribulose-5-P	C5H11O8P	229,0113	[M-H]-
Mix 1	6-Phosphogluconate	C6H13O10P	275,0168	[M-H]-
Mix 1	Succinate	C4H6O4	117,0188	[M-H]-
Mix 1	Citrate / iso-Citrate	C6H8O7	191,0192	[M-H]-
Mix 1	Acetyl-coenzyme A	C23H38N7O17P3S	808,1180	[M-H]-
Mix 1	Malate	C4H6O5	133,0137	[M-H]-
Mix 1	α-Ketoglutarate	C5H6O5	145,0137	[M-H]-
Mix 1	Fumaric acid	C4H4O4	115,0031	[M-H]-
Mix 1	Flavin adenine dinucleotide	C27H33N9O15P2	784,1493	[M-H]-
Mix 1	Guanosine 5´-diphosphate	C10H15N5O11P2	442,0165	[M-H]-
Mix 1	cis-Aconitate	C6H6O6	173,0086	[M-H]-
Mix 1	Succinyl-coenzyme A	C25H40N7O19P3S	866,1234	[M-H]-
Mix 1	Nicotinamide adenine dinucleotide reduced	C21H28N7O14P2	664,1169	[M-H]-
Mix 1	Pyruvate	C3H4O3	175,0240	[M-H]-
Mix 1	Phosphoenolpyruvate	C3H5O6P	166,9746	[M-H]-
Mix 1	Nicotinamide adenine dinucleotide	C21H27N7O14P2	662,1013	[M-H]-
Mix 2	Adenosine 5´-triphosphate	C10H16N5O13P3	505,9879	[M-H]-
Mix 2	Adenosine 5´-monophosphate	C10H14N5O7P	346,0553	[M-H]-
Mix 2	Nicotinamide adenine dinucleotide phosphate	C21H28N7O17P3	742,0676	[M-H]-
Mix 2	Nicotinamide adenine dinucleotide phosphate reduced	C21H29N7O17P3	744,0837	[M-H]-
Mix 2	Nicotinamide adenine dinucleotide reduced	C21H28N7O14P2	664,1169	[M-H]-
Mix 2	Nicotinamide adenine dinucleotide	C21H27N7O14P2	662,1013	[M-H]-
Mix 2	Thymidine	C10H14N2O5	241,0824	[M-H]-
Mix 2	2´-Deoxyadenosine	C10H13N5O3	250,0940	[M-H]-
Mix 2	2´-Deoxyinosine	C10H12N4O4	251,0780	[M-H]-
Mix 2	CMP/Cytidine 5'-monophosphate	C9H14N3O8P	322,0440	[M-H]-
Mix 2	IMP/2'Inosine 5'monophosphate	C10H13N4O8P	347,0393	[M-H]-
Mix 2	dAMP/2'Deoxyadenosine 5'monophosphate	C10H14N5O6P	330,0603	[M-H]-
Mix 2	dCMP/2'Deoxycitidine 5'monophosphate	C9H14N3O7P	306,0491	[M-H]-
Mix 2	CTP/Cytidine 5'-triphosphate	C9H16N3O14P3	481,9767	[M-H]-

Mix 2	Guanosine 5′-monophosphate (GMP)	C10H14N5O8P	362,0501	[M-H]-
Mix 2	Uridine 5′-monophosphate (UMP)	C9H13N2O9P	323,0280	[M-H]-
Mix 2	Adenosine	C10H13N5O4	266,0890	[M-H]-
Mix 2	5′-Deoxy-5′-(methylthio)adenosine (MTA)	C11H15N5O3S	296,0817	[M-H]-
Mix 2	UDP-Glucose	C15H24N2O17P2	565,0471	[M-H]-
Mix 2	GDP-Glucose	C16H25N5O16P2	604,0693	[M-H]-
Mix 2	ADP-Glucose	C16H25N5O15P2	588,0744	[M-H]-
Mix 2	cGMP	C10H12N5O7P	344,0396	[M-H]-
Mix 2	cAMP	C10H12N5O6P	328,0446	[M-H]-
Mix 2	PRPP; Phosphoribosyl pyrophosphate	C5H13O14P3	388,9439	[M-H]-
Mix 2	Uracil	C4H4N2O2	111,0194	[M-H]-
Mix 2	Uridine	C9H12N2O6	243,0617	[M-H]-
Mix 2	AICAR-monophosphate (ZMP)	C9H15N4O8P	337,0549	[M-H]-
Mix 2	L-Dihydroorotic acid	C5H6N2O4	157,0249	[M-H]-
Mix 2	dUMP	C9H13N2O8P	307,0332	[M-H]-
Mix 2	dTMP	C10H15N2O8P	321,0489	[M-H]-
Mix 3	Glutamic Acid	C5H9NO4	146,0454	[M-H]-
Mix 3	Glycine	C2H5NO2	74,0243	[M-H]-
Mix 3	Alanine	C3H7NO2	88,0399	[M-H]-
Mix 3	Serine	C3H7NO3	104,0348	[M-H]-
Mix 3	Proline	C5H9NO2	114,0556	[M-H]-
Mix 3	Valine	C5H11NO2	116,0712	[M-H]-
Mix 3	Threonine	C4H9NO3	118,0505	[M-H]-
Mix 3	Taurine	C2H7NO3S	124,0069	[M-H]-
Mix 3	Isoleucine	C6H13NO2	130,0869	[M-H]-
Mix 3	Leucine	C6H13NO2	130,0869	[M-H]-
Mix 3	Asparagine	C4H8N2O3	131,0456	[M-H]-
Mix 3	Aspartic Acid	C4H7NO4	132,0297	[M-H]-
Mix 3	Glutamine	C5H10N2O3	145,0613	[M-H]-
Mix 3	Lysine	C6H14N2O2	145,0978	[M-H]-
Mix 3	Methionine	C5H11NO2S	148,0433	[M-H]-
Mix 3	Histidine	C6H9N3O2	154,0617	[M-H]-
Mix 3	Phenylalanine	C9H11NO2	164,0712	[M-H]-
Mix 3	Arginine	C6H14N4O2	173,1039	[M-H]-
Mix 3	Tyrosine	C9H11NO3	180,0661	[M-H]-
Mix 3	Tryptophan	C11H12N2O2	203,0821	[M-H]-
Mix 3	GSH	C10H17N3O6S	306,076	[M-H]-
Mix 3	GSSG	C20H32N6O12S2	611,1442	[M-H]-
Mix 3	Ala-Gln	C8H15N3O4	216,0985	[M-H]-
Mix 3	Kynurenine	C10H12N2O3	207,077	[M-H]-
Mix 3	1-Methyl-L-histidine	C7H11N3O2	168,0774	[M-H]-

Supplementary Table 1. List of reference standards. Three standard mixtures were used for the identification of the different metabolites. The table details the m/z and the adduct considered for each metabolite.

		GO 1 vs. Control			GO 2 vs. Control			FLG vs. Control		
**Class**	**Metabolite**	**Log2 (fold-change)**	**Student's t-test (p)**	**Stat**	**Log2 (fold-change)**	**Student's t-test (p)**	**Stat**	**Log2 (fold-change)**	**Student's t-test (p)**	**Stat**
AA	Glutamic acid	0,4064	**1,39E-02**	* $p<0.05$	0,2982	**4,65E-02**	* $p<0.05$	0,0999	4,32E-01	*n.s.*
AA	Proline	0,6707	**3,99E-02**	* $p<0.05$	0,8471	**2,07E-03**	** $p<0.01$	-0,1758	2,66E-01	*n.s.*
AA	Valine	0,3303	**5,65E-03**	** $p<0.01$	0,3603	**6,19E-03**	** $p<0.01$	0,0131	9,25E-01	*n.s.*
AA	Isoleucine	0,4139	**1,81E-02**	* $p<0.05$	0,3426	**5,90E-03**	** $p<0.01$	0,1326	5,83E-01	*n.s.*
AA	Methionine	0,4036	**1,14E-02**	* $p<0.05$	0,5120	**2,12E-02**	* $p<0.05$	0,0253	8,29E-01	*n.s.*
AA	Tyrosine	0,4024	**2,59E-02**	* $p<0.05$	0,4183	**2,75E-02**	* $p<0.05$	0,0698	6,08E-01	*n.s.*
Carb acid	cis-Aconitate	0,8670	**3,81E-02**	* $p<0.05$	0,5497	**4,15E-02**	* $p<0.05$	0,4091	1,71E-01	*n.s.*
Carb acid	Malate	0,9578	**2,94E-03**	** $p<0.01$	0,8470	**3,68E-02**	* $p<0.05$	0,5297	5,15E-02	*n.s.*
Ns	Cytidine	-0,4190	**3,93E-02**	* $p<0.05$	-0,8961	**1,07E-03**	** $p<0.01$	-0,1645	2,13E-01	*n.s.*
Nt	UDP-Gluc	0,4008	**4,50E-02**	* $p<0.05$	0,5672	**1,72E-02**	* $p<0.05$	0,1623	3,65E-01	*n.s.*
AA	GSSG	1,1289	**2,50E-02**	* $p<0.05$	1,4379	1,69E-01	*n.s.*	0,7960	6,42E-02	*n.s.*
Carb acid	3-Hydroxy-glutaric acid	0,5278	**2,67E-02**	* $p<0.05$	0,0124	9,45E-01	*n.s.*	0,3363	1,11E-01	*n.s.*
Carb	Fumaric acid	0,7731	**3,02E-02**	*	0,7017	1,01E-01	*n.s.*	0,3692	2,91E-01	*n.s.*

acid				p<0.05								
Redox	NADP	0,6555	**9,91E-03**	** p<0.01		0,3896	1,44E-01	*n.s.*		0,2506	4,14E-01	*n.s.*
Redox	NADPH	0,7561	**7,86E-03**	** p<0.01		0,4449	1,75E-01	*n.s.*		0,4119	2,05E-01	*n.s.*
Vit	Folic acid	0,7563	**1,66E-02**	* p<0.05		0,5427	3,38E-01	*n.s.*		0,6385	4,86E-01	*n.s.*
Misc	CoA	0,4076	**2,84E-02**	* p<0.05		0,5406	2,58E-01	*n.s.*		-0,1935	3,27E-01	*n.s.*
CHO	2-PG. 3-PG	0,8490	1,42E-01	*n.s.*		1,6964	5,84E-03	** p<0.01		-0,7894	2,18E-01	*n.s.*
Redox	FAD	-0,0401	8,98E-01	*n.s.*		-0,4590	3,38E-02	* p<0.05		-0,0632	7,68E-01	*n.s.*
Vit	Pantothenic acid	0,5652	1,64E-01	*n.s.*		0,9607	9,98E-03	** p<0.01		0,4566	4,29E-01	*n.s.*

Supplementary Table 2. Most relevant metabolites (7d). Metabolites found to be significantly altered in the comparisons GO 1 vs. C, GO 2 vs. C, and FLG vs. C. Log2 (fold-change) and unpaired Student's t-test p-values are also displayed.

		GO 1 vs. Control			GO 2 vs. Control			FLG vs. Control		
**Class**	**Metabolite**	**Log2 (fold-change)**	**Student's t-test (p)**	**Stat**	**Log2 (fold-change)**	**Student's t-test (p)**	**Stat**	**Log2 (fold-change)**	**Student's t-test (p)**	**Stat**
AA	Pyroglutamic acid	0,434654	**4,77E-03**	** p<0.01	0,418416	**2,68E-03**	** p<0.01	0,178155	4,23E-01	*n.s.*
Carb Acid	Succinate	0,746962	**1,77E-02**	* p<0.05	0,788199	**3,36E-02**	* p<0.05	-0,24455	4,54E-01	*n.s.*
AA	N-Acetylglycine	0,83449	**4,78E-02**	* p<0.05	0,824598	6,87E-02	*n.s.*	0,178538	6,08E-01	*n.s.*
AA	Glutamic acid	0,505443	7,66E-02	*n.s.*	0,505732	**3,28E-02**	* p<0.05	-0,02125	9,41E-01	*n.s.*
AA	Valine	0,517622	8,48E-02	*n.s.*	0,603356	**1,54E-02**	* p<0.05	-0,18095	4,82E-01	*n.s.*
AA	Isoleucine	0,458623	6,20E-02	*n.s.*	0,542591	**2,99E-02**	* p<0.05	0,113737	7,18E-01	*n.s.*
AA	Leucine	0,483342	6,68E-02	*n.s.*	0,537854	**1,16E-02**	* p<0.05	0,105501	7,54E-01	*n.s.*
AA	Phenylalanine	0,530364	6,71E-02	*n.s.*	0,629838	**1,69E-02**	* p<0.05	0,087994	7,96E-01	*n.s.*
AA	Tyrosine	0,558898	1,05E-01	*n.s.*	0,633909	**1,40E-02**	* p<0.05	-0,09661	7,06E-01	*n.s.*
AA	Tryptophan	0,680052	8,02E-02	*n.s.*	0,635138	**2,91E-02**	* p<0.05	0,09982	7,42E-01	*n.s.*
AA	SAH	0,368026	2,91E-01	*n.s.*	0,783035	**3,17E-02**	* p<0.05	-0,05457	8,89E-01	*n.s.*
Carb Acid	cis-Aconitate	0,360853	4,57E-01	*n.s.*	0,97835	**2,17E-02**	* p<0.05	-0,0108	9,78E-01	*n.s.*
Carb Acid	Fumaric acid	0,592313	2,96E-01	*n.s.*	1,213934	**3,73E-02**	* p<0.05	-0,17722	7,84E-01	*n.s.*
Carb Acid	Malate	0,65046	1,54E-01	*n.s.*	1,172479	**1,36E-02**	* p<0.05	-0,08908	8,58E-01	*n.s.*
Nt	ATP	0,377626	2,63E-01	*n.s.*	0,728740	**1,59E-02**	* p<0.05	-0,10693	7,46E-01	*n.s.*

Nt	dGTP	0,395416	2,45E-01	*n.s.*	0,760112	**1,40E-02**	* p<0.05	-0,11267	7,31E-01	*n.s.*
Nt	GTP	0,644643	8,08E-02	*n.s.*	1,213540	**2,81E-03**	** p<0.01	0,084305	7,70E-01	*n.s.*
Vit	Pantothenic acid	0,464783	7,11E-02	*n.s.*	0,783348	**1,21E-02**	* p<0.05	0,310112	5,43E-01	*n.s.*
Vit	Folic acid	0,720052	1,14E-01	*n.s.*	0,728553	**2,32E-02**	* p<0.05	0,195102	6,59E-01	*n.s.*
Carb Acid	5-MTHF	1,330463	2,22E-01	*n.s.*	0,839419	2,25E-01	*n.s.*	-0,92074	**5,08E-03**	** p<0.01
Redox	FAD	-0,38785	9,31E-02	*n.s.*	-0,31876	5,25E-02	*n.s.*	-0,41279	**4,89E-02**	* p<0.05

Supplementary Table 3. Most relevant metabolites (30d). Metabolites found to be significantly altered in the comparisons GO 1 *vs*. C, GO 2 *vs.* C, and FLG *vs.* C. Log2 (fold-change) and unpaired Student's *t*-test *p*-values are also displayed.

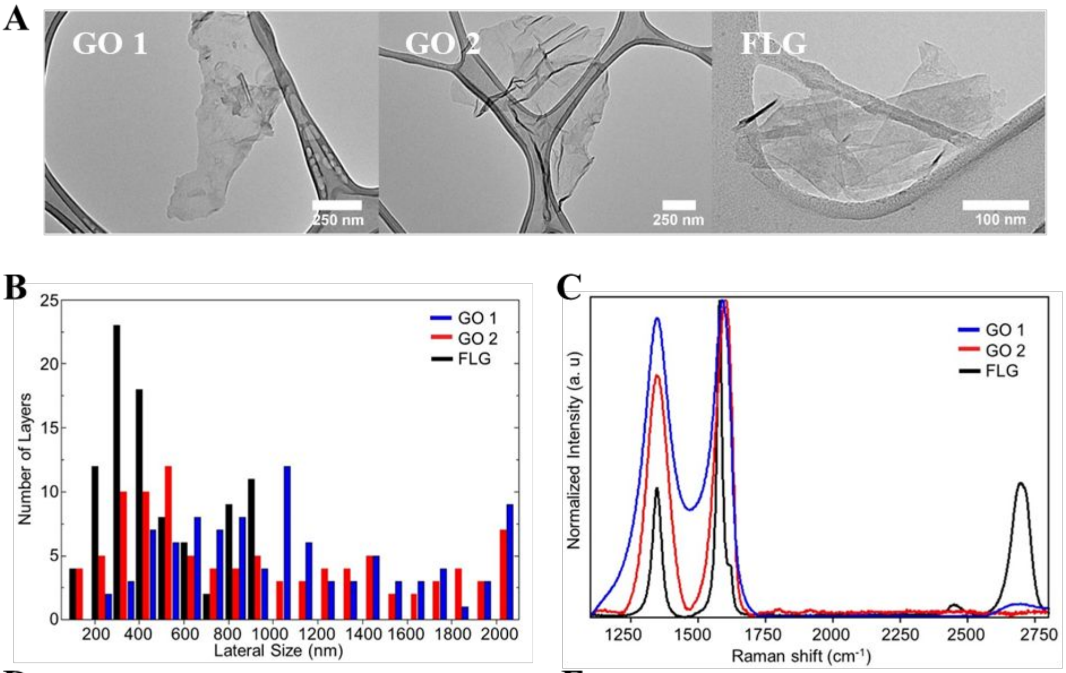

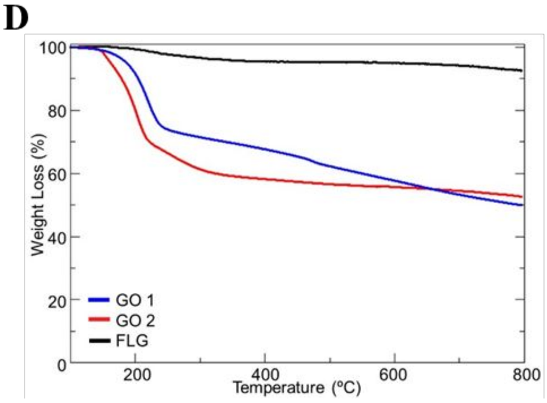

E

Sample	%C	%H	%N	%S	%O
GO 1	47.04 ±0.11	3.05 ±0.03	0.15 ±0.01	1.38 ±0.01	48.38 ±0.04
GO 2	44.24 ±0.04	2.82 ±0.06	0.50 ±0.01	2.58 ±0.01	49.86 ±0.03
FLG	91.62 ±0.43	0.79 ±0.02	0.55 ± 0.02	0.51 ± 0.02	6.53 ±0.12

Supplementary Figure 1. Characterization of GO 1, GO 2, and FLG by: HRTEM Image (A); size distribution (B); Raman spectra (C); TGA results in nitrogen atmosphere (D) and elemental analysis of nanomaterials (E).

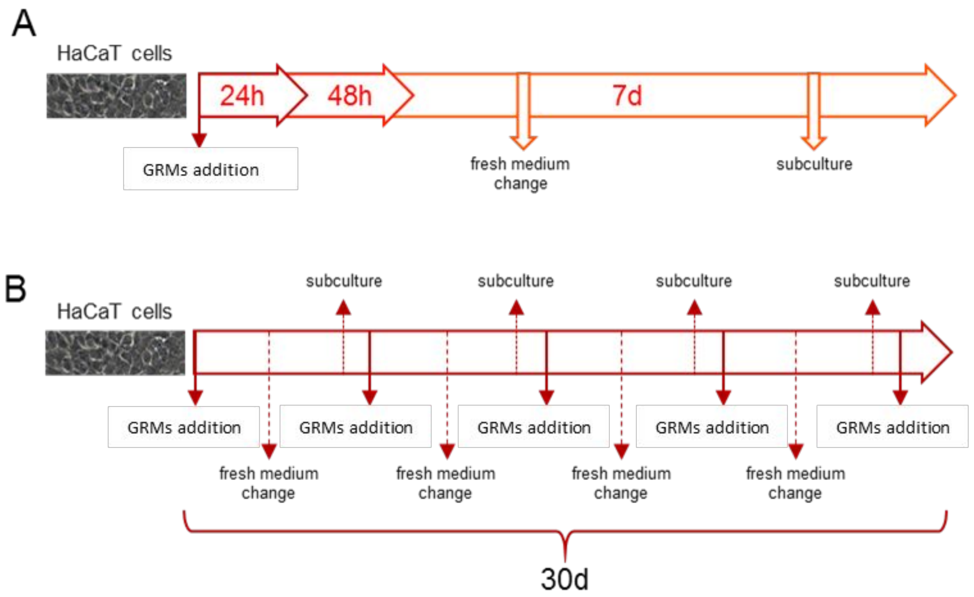

Supplementary Figure 2. GRMs exposure approach. HaCaT cells were treated 1 time per week for 1 week (A) and 30 days (B).

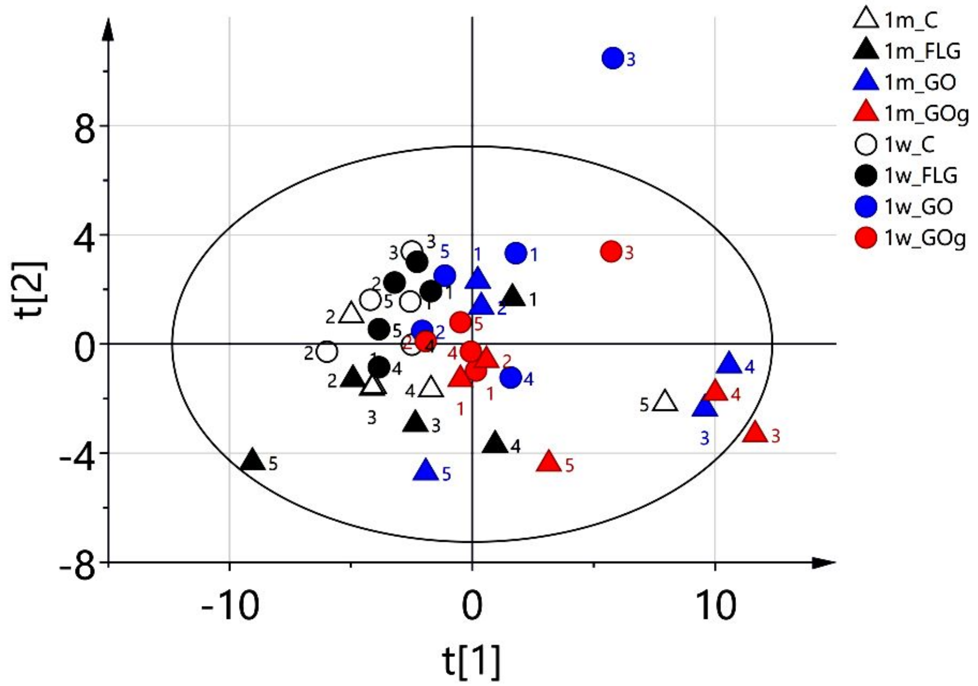

Supplementary Figure 3. Score scatter plot of the PCA model of HaCaT cells, highlighting the replica number per group (C-control; FLG-few-layer graphene; GO-Graphene oxide 1; GOg-Graphene oxide 2).

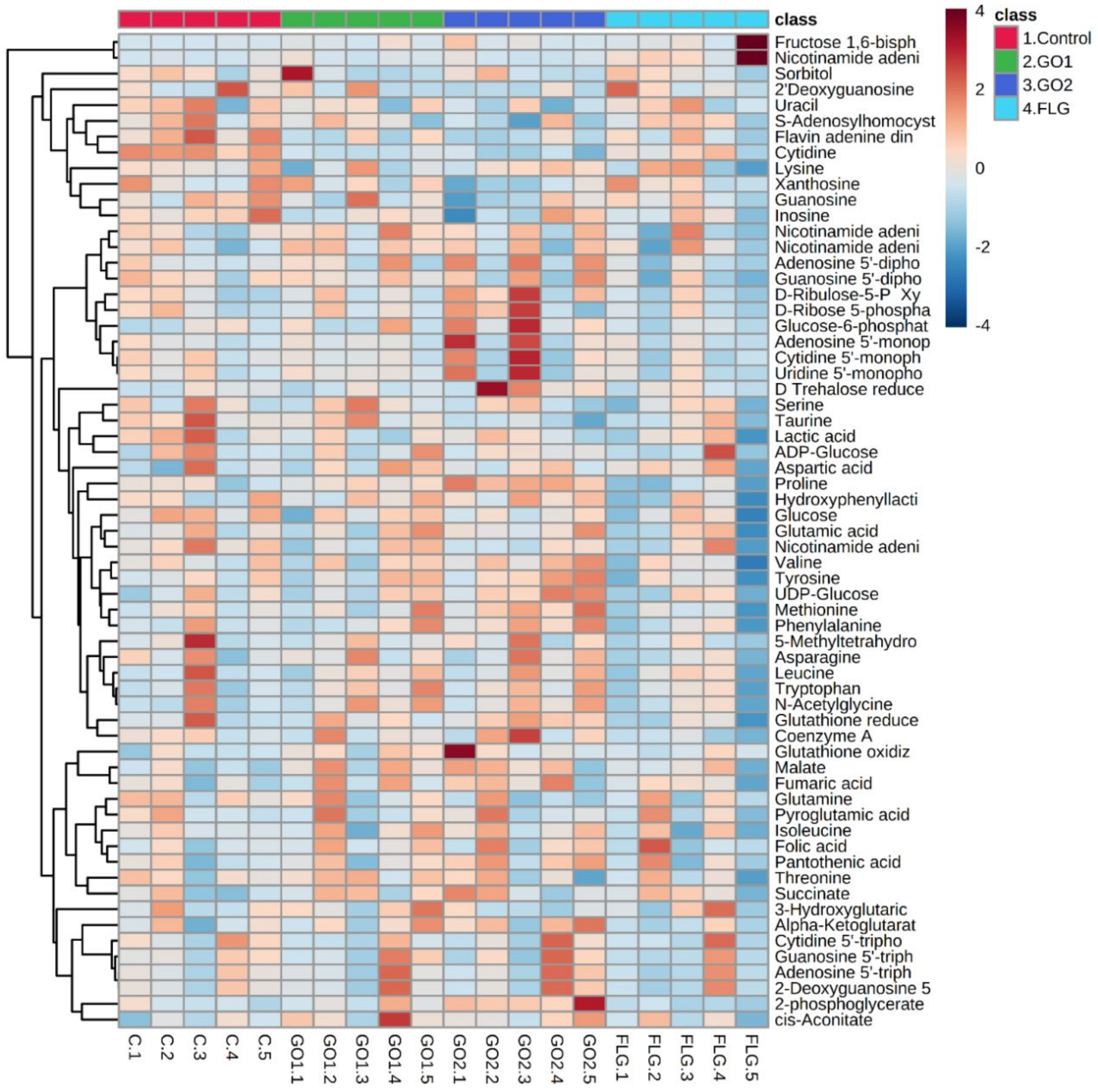

Supplementary Figure 4. Metabolites altered in HaCaT treated for 7 d with GRMs. Heatmap and clustering of metabolites corresponding to HaCaTs treated with 5μg/ml GO 1, GO 2, or FLG for 7 d (N=5).

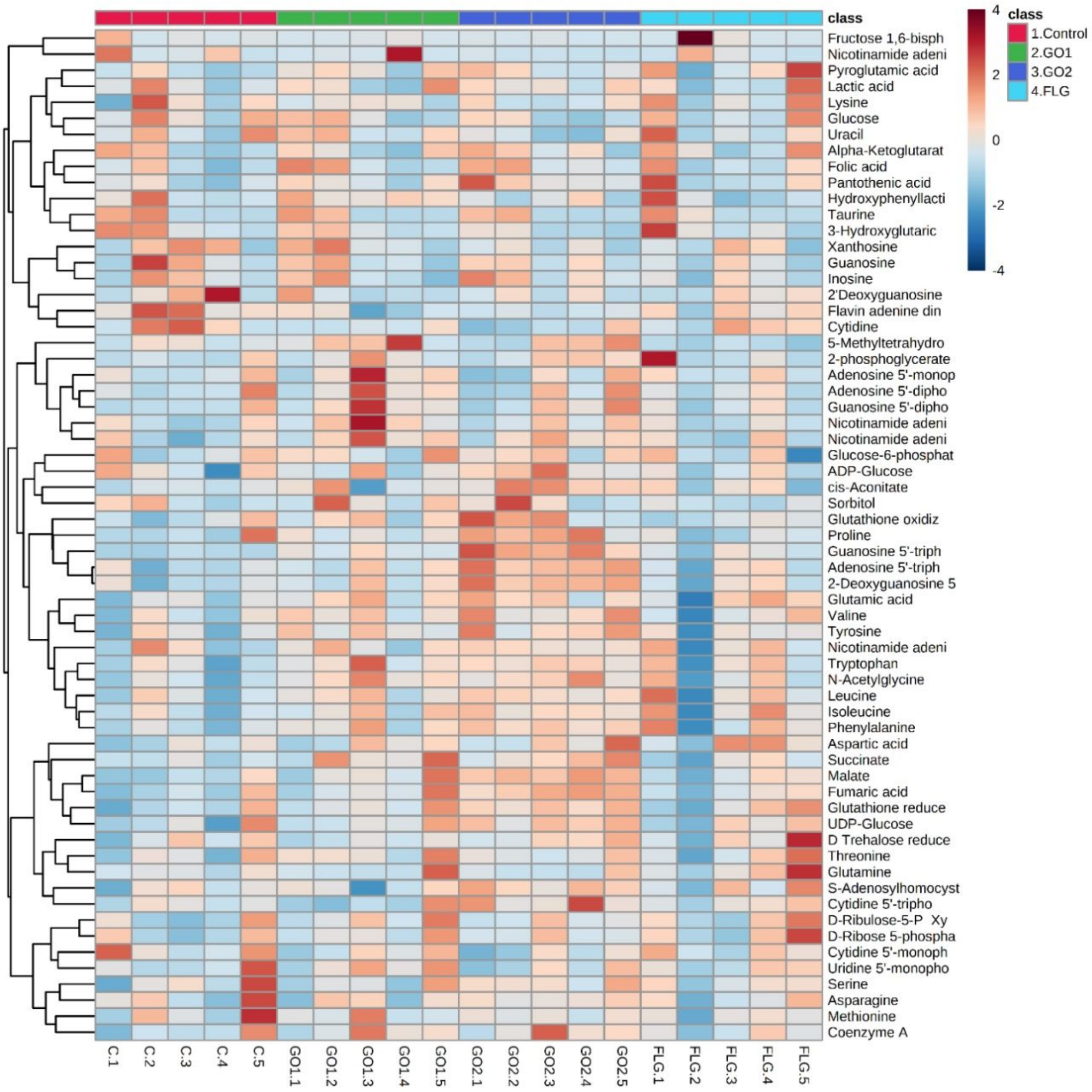

Supplementary Figure 5. Metabolites altered in HaCaT treated for 30 d with GRMs. Heatmap and clustering of metabolites corresponding to HaCaTs treated with 5µg/ml GO 1, GO 2, or FLG for 30 d (N=5).

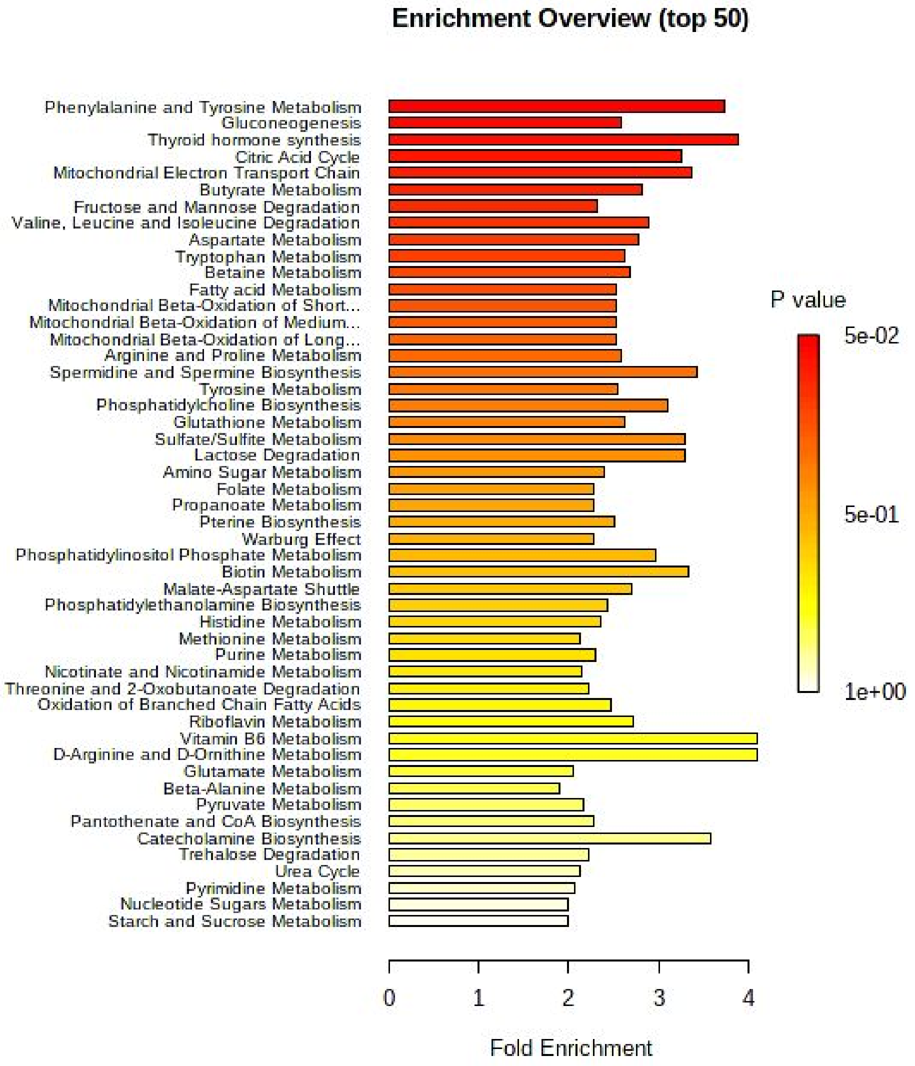

Supplementary Figure 6. Enrichment Analysis GO2 vs. control cells 30d. Summary plot for Quantitative Enrichment Analysis (QEA).

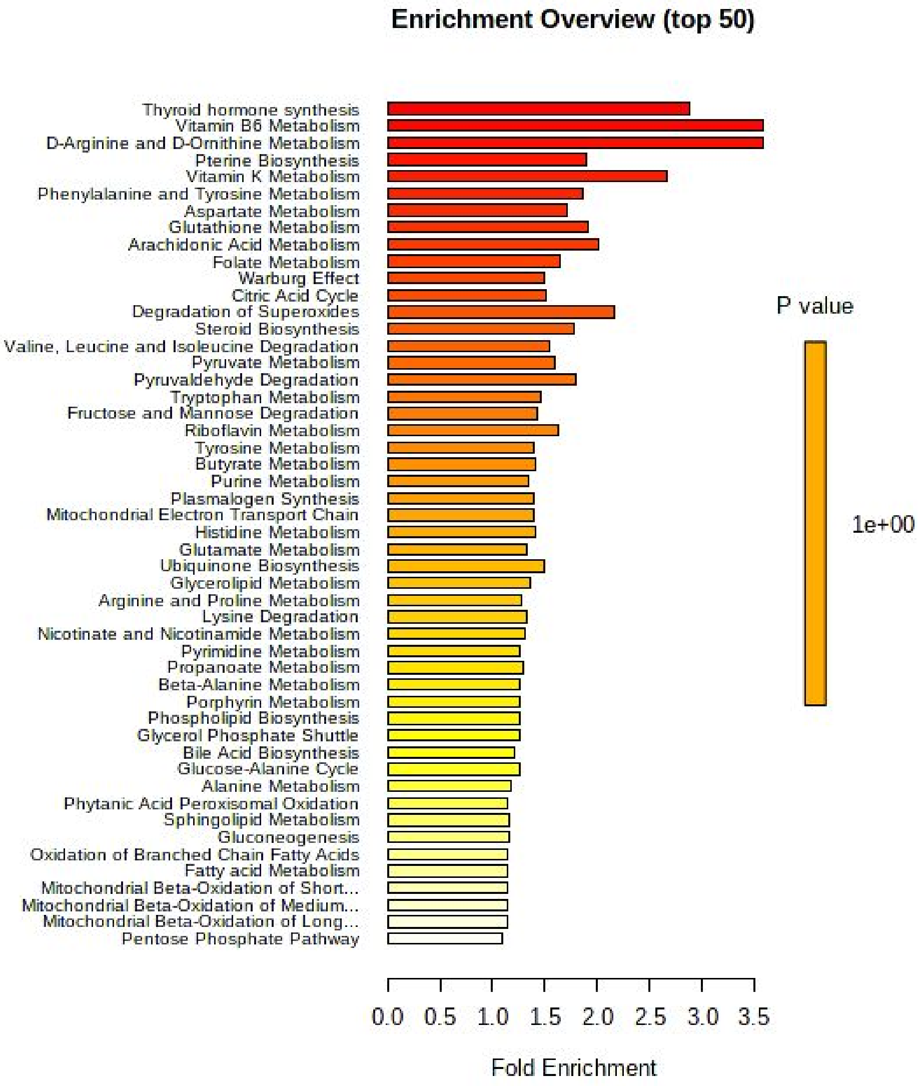

Supplementary Figure 7. Enrichment Analysis GO1 vs. control cells 30d. Summary plot for Quantitative Enrichment Analysis (QEA).

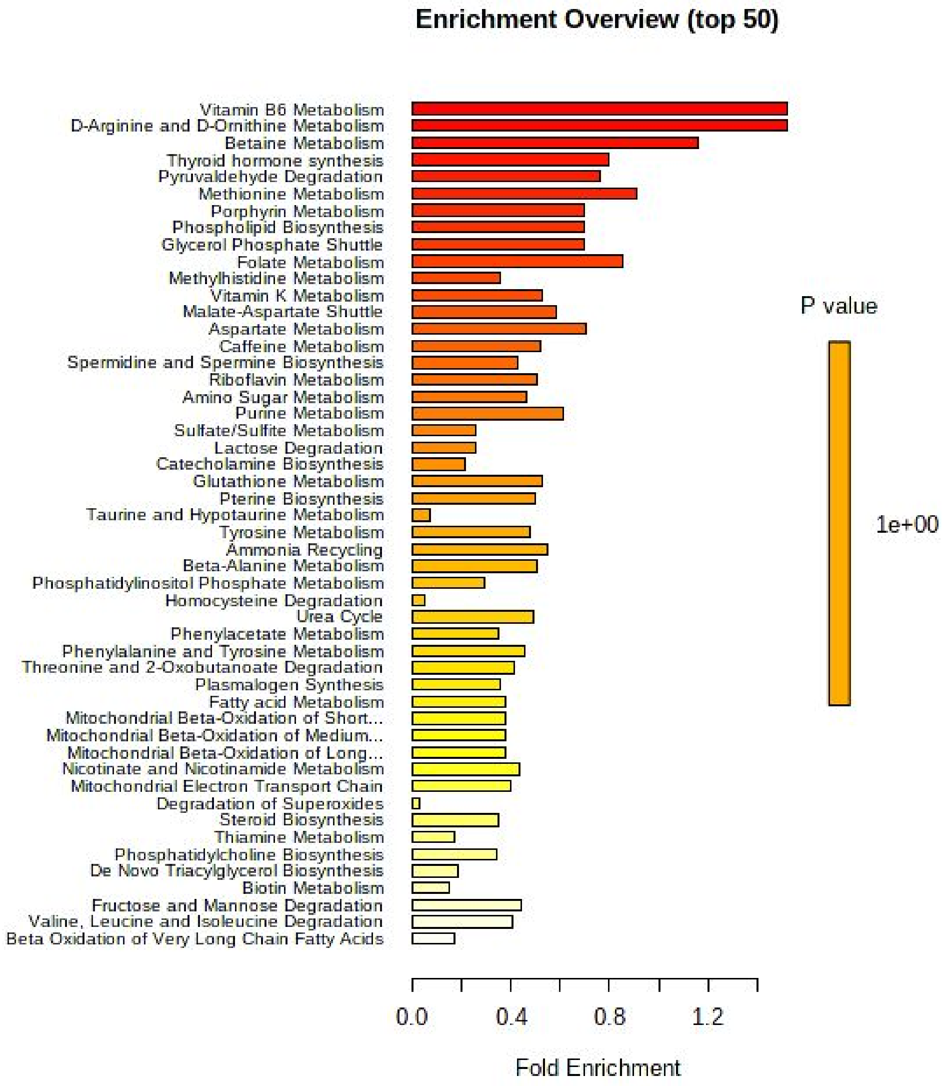

Supplementary Figure 8. Enrichment Analysis FLG vs. control cells 30d. Summary plot for Quantitative Enrichment Analysis (QEA).

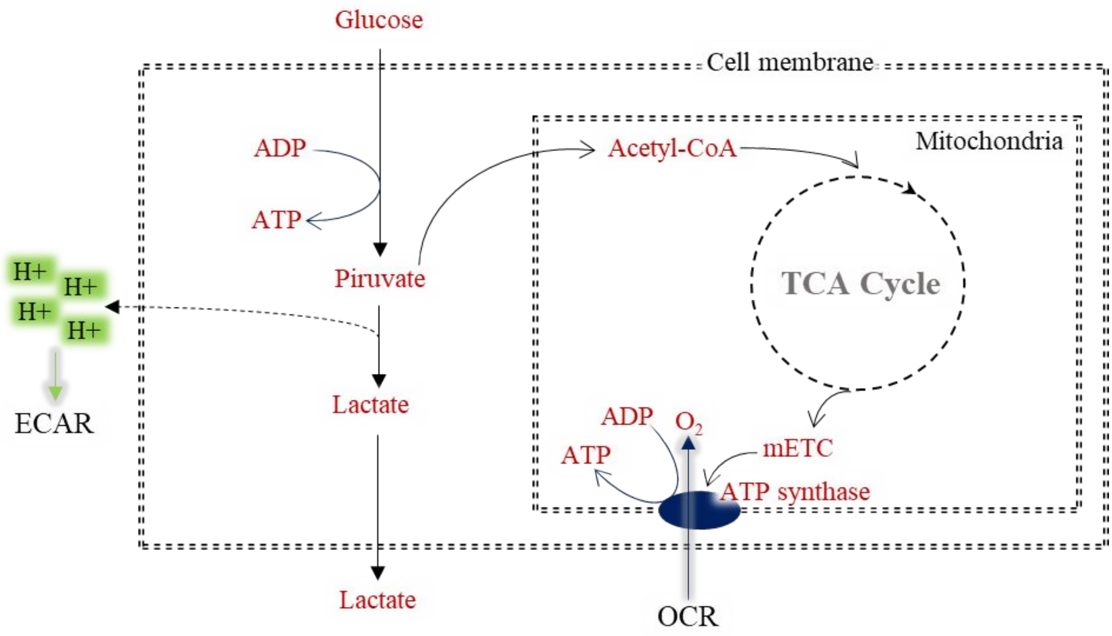

Supplementary Figure 9. General metabolism scheme and Seahorse XFp source measurements. The ATP synthase oxygen consumption mainly measures mitochondrial respiration. Glycolysis is quantified by measuring ECAR of the surrounding media.

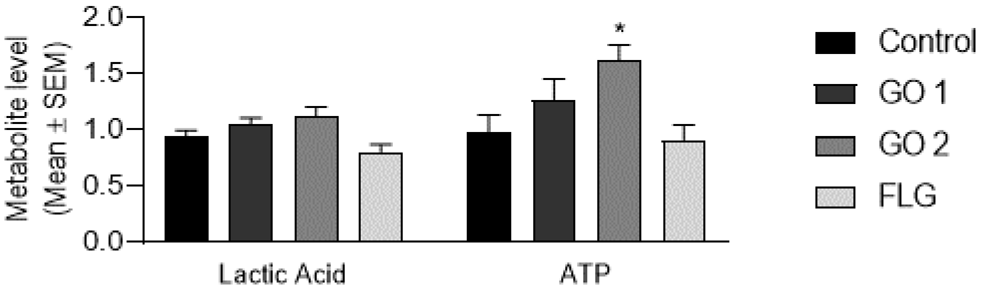

Supplementary Figure 10. The effect of GRMs on cell metabolism. Normalized lactic acid and ATP levels in HaCaTs treated with GO 1, GO 2, or FLG for 30 d (mean ± SEM; N=5).

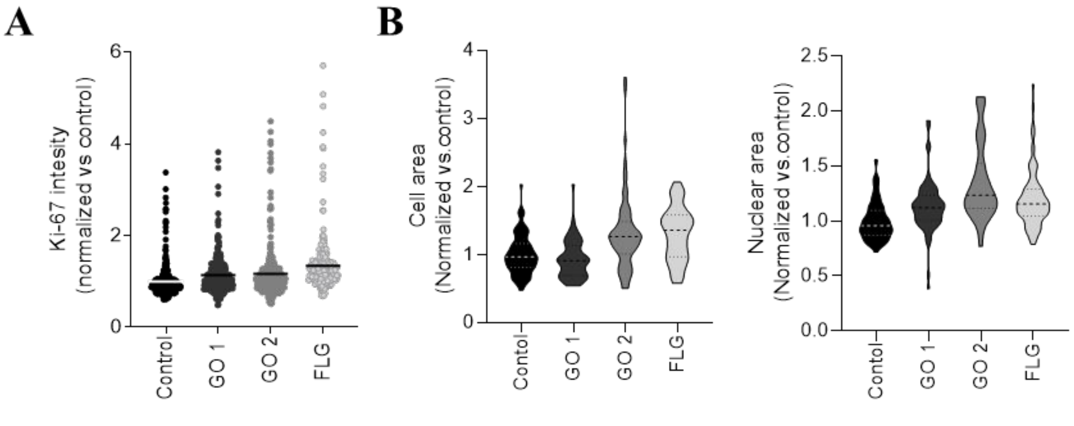

Supplementary Figure 11. The effect of GRMs on cell proliferation and phenotype. Proliferation measured as Ki-67 intensity per cell (a) and the nuclear and cell area (b) in HaCaTs treated with GO 1, GO 2, or FLG for 30 d (>50 cells) (normalized vs. control).
